# New Orleans Medical Register

**Published:** 1852-05

**Authors:** 


*N. O. Med. Register.*

The New Orleans Medical Register, edited by A. Forster Axson, M.D., is a neat little work of 12 pages, containing a variety of interesting original and selected matter. We extend to the editor the right hand of fellowship, and wish for him and his little one the success always due to merit. J.

